# Validation of the Prognostic Role for Surgical Treatment in Stage II Intrahepatic Cholangiocarcinoma: A SEER Population-Based Study

**DOI:** 10.3390/jcm12020675

**Published:** 2023-01-14

**Authors:** Shuaiwu Luo, Linquan Wu, Min Li, Jiakun Wang, Cong Wang, Jun Yang, Ligan Zhang, Jin Ge, Chi Sun, Enliang Li, Jun Lei, Fan Zhou, Wenjun Liao

**Affiliations:** 1 Department of Clinical Medicine, Nanchang University, No. 999, University Avenue, Nanchang 330006, China; 2 Department of General Surgery, The Second Affiliated Hospital of Nanchang University, No. 1, Minde Road, Nanchang 330006, China

**Keywords:** liver cancer, surveillance epidemiology and end results program epidemiology, propensity scores, prognosis

## Abstract

Background: This study aimed to determine the role of surgical treatment in patients with stage II intrahepatic cholangiocarcinoma (iCCA). Methods: Data were extracted from the Surveillance, Epidemiology, and End Results (SEER) database. We divided stage II iCCAs into solitary tumors with vascular invasion (T2sN0M0) and multiple tumors with/without vascular invasion (T2mN0M0) according to the criteria of AJCC v.8. The Kaplan–Meier method and log-rank test were used to evaluate differences in overall survival (OS). We performed two propensity score-matching analyses with (PSM2) or without (PSM1) surgical treatment. Results: 667 and 778 iCCA patients with stage II and IIIB were recruited. After PSM2, there was no survival difference in stage II iCCA patients in hypothetical conditions with similar surgical proportions (*p* = 0.079). However, OS was significantly worse in patients with T2mN0M0 than T2sN0M0 when the actual surgical proportion existed after PSM1 (*p* < 0.001). OS was similar between T2mN0M0 and IIIB regardless of whether PSM1 (*p* = 0.907) or PSM2 (*p* = 0.699) was performed. The surgical treatment was verified to associate with prognosis. Conclusions: The survival benefit by surgical treatment was existed in Stage II but not in Stage IIIB iCCA patients. The OS for T2mN0M0 will approach that of T2sN0M0 if the surgical proportion is gradually increased.

## 1. Introduction

Intrahepatic cholangiocarcinoma (iCCA) is the second most common malignant tumor of the liver, accounting for 10% to 15% of all liver cancers [1,2]. Although the annual incidence of iCCA has remained low during recent decades, it is dramatically increasing worldwide [3]. The prognosis of iCCA is poor because this tumor usually presents in its advanced stages [4,5]. Several independent factors are associated with worse long-term survival, including the presence of vascular invasion, distant metastasis, regional lymph node metastasis, and multiple tumors [6]. Thus, accurate staging to stratify patients with iCCA for proper disease management is important.

In 2018, the eighth edition of the American Joint Committee on Cancer (AJCC) staging system (AJCC v.8) proposed a new standard for stratifying patients with iCCA, predicting the prognosis, and guiding treatment decisions [7]. In AJCC v.8, T2a (solitary tumor with vascular invasion) and T2b (multiple tumors with or without vascular invasion) were merged into T2, and T2N0M0 is defined as stage II. In several large cohort studies of the ICCA staging system in AJCC v.7, it was found that vascular invasion and multifocal tumors had similar effects on the survival and prognosis of ICCA [8,9,10]. Therefore, the T2 category is modified in AJCC v.8 to reflect the equivalent prognostic value of vascular invasion and tumor multifocality, and level of evidence is II (that is, the available evidence is obtained from at least one large, well-designed, and well-conducted study in appropriate patient populations with appropriate endpoints and with external validation). This conclusion was also confirmed by two studies [11,12].

However, we found that the survival difference was existed in stage II iCCA patients in clinical practice, especially in patients with multiple tumors with vascular invasion. It seemed like a shorter overall survival (OS) time existed in these patients compared with patients with solitary tumors. This was contrary to the conclusion of AJCC v.8. To identify the cause of this problem and explore the controversial issues surrounding stage II disease, we used the Surveillance, Epidemiology, and End Results (SEER) database in the United States (US) to investigate the prognostic differences between multifocal disease and solitary lesions in patients with stage II iCCA in an effort to provide a reasonable explanation for this controversy.

## 2. Materials and Methods

### 2.1. Study Design and Patient Selection

In this study, stage II iCCAs were classified into the following two groups: (1) solitary tumors with vascular invasion and without lymph node or distant metastasis (T2sN0M0) and (2) multiple tumors with/without vascular invasion and without lymph node or distant metastasis (T2mN0M0). The primary objective of this study was to confirm whether the classification of T2N0M0 in AJCC v.8 is accurate. The secondary objective was to examine the previous classification of T2mN0M0 as stage IVa and determine the survival prognosis.

Data were obtained from the SEER database, in which all patients with primary iCCA from 2004 to 2015 were identified. We used the SEER-18 database; its data come from 18 United States regions, accounting for about 27.8 percent of the total population. The inclusion criterion was a diagnosis of iCCA classified with the International Classification of Diseases for Oncology, third edition codes (site: C22.0, C22.1 and histology/behavior: 8160/3 [cholangiocarcinoma]). TNM information was retrieved based on the following codes: derived AJCC T (sixth edition, 2004–2015; seventh edition, 2010–2015), derived AJCC N (sixth edition, 2004–2015; seventh edition, 2010–2015), derived AJCC M (sixth edition, 2004–2015; seventh edition, 2010–2015), and CS extension (2004–2015). In the project of CS extension (2004–2015), the codes corresponding to each group were as follows: T2sN0M0 (200, 220, 240, 250, 260, 270, 520, 631), T2mN0M0 (300, 400, 420, 450, 455, 460, 465, 475, 632, 650, 675), and stage IIIB (580, 620, 660, 665, 700, 750, 755, 760, 800). The specific meaning of CS extension (2004–2015) is shown in Supplementary Table S1. Surgical treatments included various types of hepatectomy and transplantation. The exclusion criteria were (1) another primary tumor; (2) diagnosis within 1 month before death (based on diagnosis reported on the death certificate or determined at autopsy) because the survival time was recorded in months, not days; and (3) death of causes other than iCCA.

### 2.2. Statistical Analysis

The 1-, 3-, and 5-year overall survival (OS) of each group was estimated according to the Kaplan–Meier method, and survival curves were compared using the log-rank test. For stage II iCCAs, univariate and multivariable Cox proportional hazard models were used to investigate the association of demographics and clinical characteristics with OS. Categorical variables were compared using the chi-square test or Fisher’s exact test, and continuous variables were compared using the t-test. Propensity score matching (PSM) was performed using 1:1 fixed-ratio nearest-neighbor matching between T2sN0M0 and T2mN0M0. This is a technique that simulates a randomized experimental study in an observational data set in order to estimate a causal effect for key variables. So, we could use PSM to identify the prognostic role of independent factors in iCCA patients. In this study, we performed PSM twice (PSM1 and PSM2) for each group. The matched confounding factors for the PSM1 analysis included age, race, sex, and tumor size, and surgical treatment was added for the PSM2 analysis. In addition, a subgroup analysis according to sex (female and male), age (<70 and ≥70 years) and types of surgery (Wedge or segmental resection, Lobectomy and Extended lobectomy) was performed. PSM was also performed using 1:1 fixed-ratio nearest-neighbor matching between T2mN0M0 and stage IIIB (including T4N0M0 and any TN1M0). All P values were two-tailed, and values of <0.05 were considered statistically significant. All statistical analyses were performed using SPSS version 26.0.0 (IBM Corp., Armonk, NY, USA).

## 3. Results

### 3.1. Patient Characteristics

The clinical characteristics of all enrolled patients with T2N0M0 iCCAs (according to AJCC v.8) are summarized in Table 1. A total of 667 patients (316 [47.4%] men and 351 [52.6%] women; median [interquartile range] age, 65 [56 years–72 years] years) met the inclusion criteria. The study population comprised 233 (34.9%) patients with T2sN0M0 iCCA and 434 (65.1%) patients with T2mN0M0 iCCA (Table 1). The tumor size was >5 cm in most patients (57.6%). In addition, the proportions of patients with T2sN0M0 and T2mN0M0 whose tumor size was ≤5 cm significantly differed (34.3% vs. 25.1%, respectively; *p* = 0.026). However, there was no significant difference in age, ethnicity, or sex between the two groups, and most patients with iCCA were Caucasians (75.3%). Among 233 patients with T2sN0M0, 51.1% underwent surgical treatment. However, among 434 patients with T2mN0M0, only 28.6% underwent surgical treatment (*p* < 0.001).

The clinical characteristics of patients with T2mN0M0 and stage IIIB are summarized in Table 2. The proportions of patients with T2mN0M0 and stage IIIB whose tumor size was ≤5 cm significantly differed (25.1% vs. 29.3%, respectively; *p* = 0.034). However, there was no significant difference in age, ethnicity, sex, or surgical treatment between the two groups.

### 3.2. OS Analysis between T2sN0M0 and T2mN0M0

**Figure 1:**
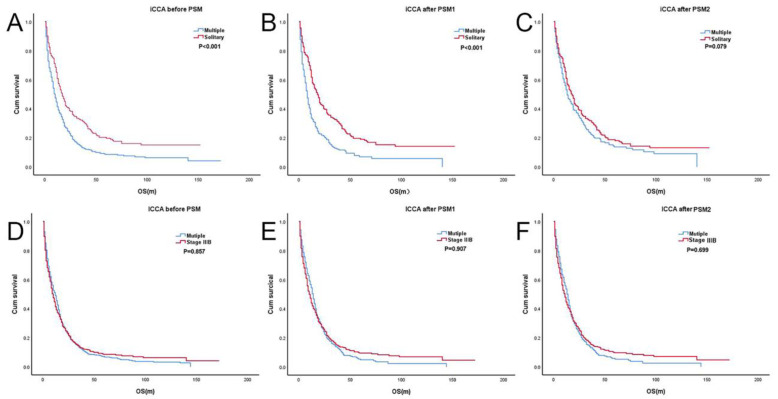
Kaplan-Meier survival curve of iCCAs. (**A**) T2sN0M0 vs. T2mN0M0 before PSM; (**B**) T2sN0M0 vs. T2mN0M0 after PSM1; (**C**) T2sN0M0 vs. T2mN0M0 after PSM2; (**D**) Stage IIIB vs. T2mN0M0 before PSM; (**E**) Stage IIIB vs. T2mN0M0 after PSM1. (**F**) Stage IIIB vs. T2mN0M0 after PSM2.Solitary: T2sN0M0, Multiple: T2mN0M0.

Before PSM analysis, the OS at 1, 3, and 5 years among patients with T2mN0M0 (41.4%, 13.4%, and 10.0%, respectively) was significantly lower than that among patients with T2sN0M0 (60.8%, 32.1%, and 22.0%, respectively) (log-rank *p* < 0.001; hazard ratio [HR], 1.61; 95% confidence interval [CI], 1.36–1.9) (Figure 1A). The survival prognosis of T2mN0M0 was significantly worse than that of T2sN0M0. In PSM1 analysis without variable adjustment for surgical proportion, among 404 patients with iCCA were recruited, 202 had T2sN0M0 and 202 had T2mN0M0 (Table 1). The log-rank test also revealed that T2mN0M0 had significantly worse OS than T2sN0M0 (*p* < 0.001; HR, 1.89; 95% CI, 1.52–2.37) (Figure 1B). That means the survival differences existed in iCCA patients with Stage II when actual surgical proportion in this stage was used. However, after PSM2 analysis, 396 patients with iCCA were included, and the 1-, 3-, and 5-year OS rates were 60.9%, 31.8%, and 20.6% in T2sN0M0 and 53.0%, 22.6%, and 16.6% in T2mN0M0, respectively (*p* = 0.079; HR, 1.22; 95% CI, 0.98–1.52) (Figure 1C). These results indicate that when the proportion of patients who underwent surgical treatment was similar, there was no survival difference between the two groups. It seemed that the survival differences disappeared in iCCA patients with Stage II when a hypothetical condition with similar surgical proportion in this stage was used.

### 3.3. Subgroup Analysis According to Age (T2sN0M0 and T2mN0M0)

**Figure 2:**
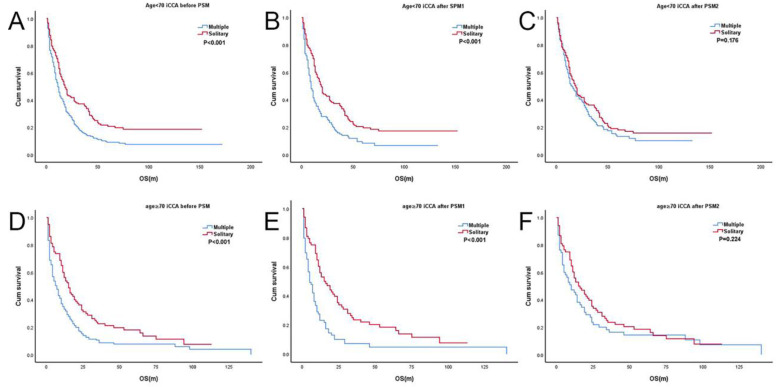
Kaplan-Meier survival curve of T2sN0M0 vs. T2mN0M0 in iCCAs stratified by age. (**A**) T2sN0M0 vs. T2mN0M0 in age < 70 years group before PSM; (**B**) T2sN0M0 vs. T2mN0M0 in age < 70 years group after PSM1; (**C**) T2sN0M0 vs. T2mN0M0 in age < 70 years group after PSM2; (**D**) T2sN0M0 vs. T2mN0M0 in age ≥ 70 years group before PSM; (**E**) T2sN0M0 vs. T2mN0M0 in age ≥ 70 years group after PSM1; (**F**) T2sN0M0 vs. T2mN0M0 in age ≥ 70 years group after PSM2. Solitary: T2sN0M0, Multiple: T2mN0M0.

Before PSM, 436 and 231 patients were <70 and ≥70 years old, respectively (Supplementary Table S2). In both the <70-year age group (*p* < 0.001; HR, 1.58; 95% CI, 1.28–1.96) and ≥70-year age group (*p* < 0.001; HR, 1.66; 95% CI, 1.25–2.21), T2mN0M0 showed worse survival than T2sN0M0 (Figure 2A,D). After PSM1, 267 patients aged <70 years and 137 patients aged ≥70 years were enrolled (Supplementary Table S2). Similar to the results before PSM, the log-rank tests indicated that the OS of T2mN0M0 was significantly worse than that of T2sN0M0 in both age groups: <70 years (*p* < 0.001, HR, 1.77; 95% CI, 1.34–2.32) and ≥70 years (*p* < 0.001; HR, 2.25; 95% CI, 1.52–3.32) (Figure 2B,E). After PSM2, 276 and 123 patients remained in the age groups (Supplementary Table S2). However, there were no statistically significant differences in OS between T2sN0M0 and T2mN0M0 in both groups (<70 years: *p* = 0.176; HR, 1.20; 95% CI, 0.92–1.57 and ≥70 years: *p* = 0.224; HR, 1.28; 95% CI, 0.86–1.90) (Figure 2C,F).

### 3.4. Subgroup Analysis According to Sex (T2sN0M0 and T2mN0M0)

**Figure 3:**
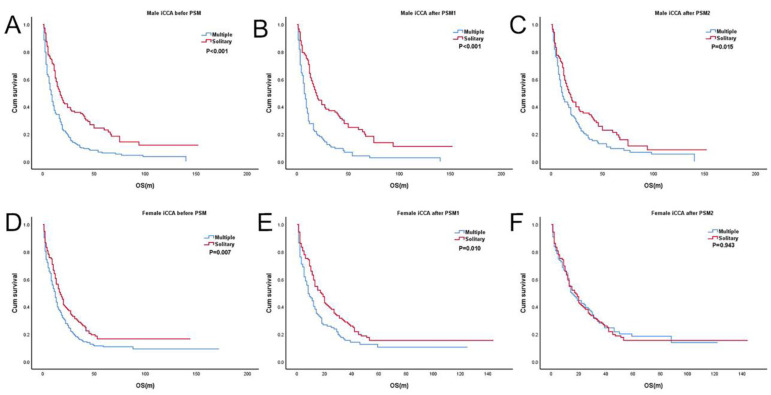
Kaplan-Meier survival curve of T2sN0M0 vs. T2mN0M0 in iCCAs stratified by sex. (**A**) T2sN0M0 vs. T2mN0M0 in male group before PSM; (**B**) T2sN0M0 vs. T2mN0M0 in male group after PSM1; (**C**) T2sN0M0 vs. T2mN0M0 in male group after PSM2; (**D**) T2sN0M0 vs. T2mN0M0 in female group before PSM; (**E**) T2sN0M0 vs. T2mN0M0 in female group after PSM1; (**F**) T2sN0M0 vs. T2mN0M0 in female group after PSM2. Solitary: T2sN0M0, Multiple: T2mN0M0.

The entire study population comprised 351 women and 316 men before PSM (Table 3). T2mN0M0 was associated with worse OS than T2sN0M0 in women (*p* = 0.007; HR, 1.39; 95% CI, 1.09–1.76) and men (*p* < 0.001; HR, 1.90; 95% CI, 1.49–2.42) (Figure 3A,D). After PSM1, 206 women and 198 men remained (Table 3). Similar to the results prior to the PSM analysis, T2mN0M0 had worse survival than T2sN0M0 in both sexes (women: *p* = 0.010; HR, 1.51; 95% CI, 1.10–2.06 and men: *p* < 0.001; HR, 2.38; 95% CI, 1.73–3.27) (Figure 3B,E). After PSM2, 208 women and 188 men remained (Table 3). T2sN0M0 and T2mN0M0 had similar OS in women (*p* = 0.943; HR, 0.99; 95% CI, 0.73–1.35) (Figure 3F). In men, however, T2mN0M0 still showed worse survival than T2sN0M0 (*p* = 0.015; HR, 1.47; 95% CI, 1.08–2.01) (Figure 3C). These results indicate that even under the condition of a similar proportion of patients undergoing surgical treatment, the prognosis of T2mN0M0 is still worse in men.

### 3.5. Subgroup Analysis According to Types of Surgery (T2sN0M0 and T2mN0M0)

The cohort included 69 patients who underwent wedge or segmental resection, 86 patients who underwent lobectomy, and 36 who patients underwent extended lobectomy (Supplementary Table S3). There was no significant difference in age, race, gender, and tumor size between T2sN0M0 and T2mN0M0 in each subgroup. In the wedge or segmental resection group (*p* = 0.110), lobectomy group (*p* = 0.603) and extended lobectomy group (*p* = 0.382), T2sN0M0 and T2mN0M0 had similar OS (Supplementary Figure S1).

### 3.6. OS Analysis between T2mN0M0 and Stage IIIB

Before PSM, the 1-, 3-, and 5-year OS rates were 41.4%, 13.4%, and 8.6% in T2mN0M0 and 48.9%, 12.8%, and 6.7% in stage IIIB, respectively (*p* = 0.857; HR, 1.01; 95% CI, 0.89–1.15) (Figure 1D). There was no survival difference between the two groups. The survival of these two groups was similar after PSM1 analysis was performed (*p* = 0.907; HR, 0.99; 95% CI, 0.85–1.16) (Figure 1E). After PSM2, the OS at 1, 3, and 5 years still showed no survival difference between T2mN0M0 (44.5%, 15.5%, and 9.7%, respectively) and stage IIIB (52.7%, 12.9%, and 5.2%, respectively) (log-rank *p* = 0.699; HR, 0.699; 95% CI, 0.83–1.14) (Figure 1F). It seemed that the survival benefit of surgical treatment in stage IIIB was unobvious.

### 3.7. Cox Regression Analysis for T2sN0M0 and T2mN0M0

In the univariate analysis, surgical treatment was associated with better OS, while older age (≥70 years), larger tumor size (>5 cm), and multiple lesions were significantly associated with worse OS (Table 4). Race were not associated with OS. The multivariable Cox regression showed that age (≥70 years) (HR, 1.208; 95% CI, 1.005–1.453; *p* = 0.040), surgical treatment (HR, 0.304; 95% CI, 0.248–0.372; *p* < 0.001), and multiple lesions (HR, 1.236; 95% CI, 1.021–1.496; *p* = 0.030) were independently associated with OS (Table 4).

## 4. Discussion

The AJCC v.8 provides a novel staging system for iCCA, and the staging of iCCA has thus gained significant attention [13]. However, the OS in stage II is debated. Lamarca et al. [14] recommended the reclassification of iCCA with multiple lesions into a more advanced category. However, some studies [11,12] have shown that both T2sN0M0 and T2mN0M0 have similar survival. Accurate disease staging is critical for the selection of proper treatments and the prediction of treatment outcomes. Thus, we investigated the differences between patients with T2sN0M0 and T2mN0M0 iCCA in terms of (1) demographic and clinical characteristics and (2) OS using a large United States population-based cancer database.

Before PSM and after the PSM1 analysis in the present study, the survival of patients with T2sN0M0 was significantly better than that of patients with T2mN0M0. However, after the PSM2 analysis (which involved a similar percentage of surgical treatment), no significant difference in OS was noted between the T2mN0M0 group and the T2sN0M0 group. Obviously, the key variable in these groups is surgical treatment. So we believe that this discrepancy was mainly caused by the difference in the ratio of surgical treatment between the two analyses. The rate of surgical treatment is higher for T2sN0M0 than for T2mN0M0 iCCA, and this was confirmed in our study. Additionally, it has been proven that surgical treatment can improve the survival of patients with iCCA [15]. However, the resectability of T2mN0M0 iCCAs is unclear. The NCCN guidelines [16] state that multifocal liver disease is a contraindication to resection but that resection can be considered in highly selected patients with limited multifocal disease. These highly selected patients often benefit from surgical treatment. A unified standard of the indications for surgical treatment of multifocal iCCA is lacking among other guidelines [11,17,18,19,20,21]. As a result, the surgical treatment rate of T2mN0M0 is much lower than that of T2sN0M0. Interestingly, our study showed that when the surgical treatment ratios of T2mN0M0 and T2sN0M0 were theoretically similar, there was no difference in the prognosis between the two groups. In other words, patients with T2sN0M0 iCCA may substantially benefit from surgical treatment. Moreover, a study by Spolverato et al. [22] also confirmed that liver resection can be safely performed for patients with multifocal iCCA. Therefore, the proportion of surgical treatment of T2mN0M0 iCCA has the potential to increase, and this increase may improve the OS prognosis of these patients. Under the current condition that the surgical treatment rate of T2mN0M0 cannot be improved to the same as that of T2sN0M0, T2mN0M0 should be classified as a more advanced stage.

We also compared T2mN0M0 and stage IIIB iCCAs. Our study indicated that before PSM and after the PSM1/PSM2 analyses, the survival of T2mN0M0 was similar to that of stage IIIB iCCAs. This result indicates that the survival benefit of surgical treatment in stage IIIB is unobvious. It is obvious that surgical treatment is not recommended as a priority in guidelines. In addition, it is worth noting that for men, even if the proportion of surgical treatment is similar (after PSM2 analysis), survival is still worse for T2mN0M0 than T2sN0M0 iCCA. This phenomenon indicates the presence of sex-related survival differences in T2N0M0 iCCA. Georgios et al. [23] suggested that the male sex is a poor predictor of survival in patients with iCCA. This phenomenon is not only unique to iCCA but can also be observed in other primary liver cancers, such as hepatocellular carcinoma. Yin et al. [24] found that sex was independently associated with OS in patients with multiple hepatocellular carcinoma. These findings indicate that sex affects the biological behavior of liver cancer, resulting in different outcomes. Therefore, even if surgical treatment is performed, it is necessary to consider the possibility of a poor prognosis for men with T2mN0M0 iCCA.

In terms of surgical treatment, we studied whether survival varies based on different types of surgery. A study by Angelico et al. showed that active surgical procedures are reasonable in selected iCCA patients with vascular involvement, and different types of surgery give patients acceptable surgical results. However, it is not clear whether T2mN0M0 iCCAs can gain similar surgical benefits to T2sN0M0 iCCAs in different types of surgery. Our study indicated that T2mN0M0 and T2sN0M0 had similar OS in a wedge or segmental resection, lobectomy, and extended lobectomy. This showed that among the three types of surgery, T2mN0M0 iCCAs can obtain comparable surgical benefits as T2sN0M0 iCCAs.

Our multivariate analysis showed that age, surgical treatment, and multiple lesions were significantly associated with OS. This was also suggested by Raoof et al. [6], who reported that older age and multiple lesions were associated with worse OS. Data from a large multi-institutional study also corroborated the effects of tumor number and age [8]. In addition, many studies have confirmed the beneficial effects of surgical treatment on survival in patients with iCCAs [19,22,25,26,27].

Our study cleverly used PSM twice to make a lot of meaningful findings. Our study confirmed that the surgical benefits of T2mN0M0 are comparable to T2sN0M0, and the same results were obtained even under different types of surgery. More importantly, our study pointed out that the prognosis of T2mN0M0 was worse than T2sN0M0, and the key factor leading to the poor prognosis of T2mN0M0 was its lower rate of surgical treatment. All these results are different from a study by Linlin Yin et al. [28]. However, the current study had three main limitations that should be considered. First, was a retrospective study and therefore had inherent defects. Second, the SEER 18 Database does not provide granular clinical data that can affect the prognosis, such as the severity of underlying liver disease or related health conditions. Finally, the SEER database only collects data on the population of the United States. As the incidences and mortality of iCCA are different in different geographical regions, global multicenter research is our next direction of research.

## 5. Conclusions

The prognosis of T2N0M0 with multiple tumors and T2N0M0 with a solitary tumor is similar only when the proportion of patients undergoing surgery is similar. In fact, the prognosis of T2N0M0 with multiple tumors is significantly worse than that of T2N0M0 with a solitary tumor and similar to that of stage IIIB iCCA. However, patients who have T2N0M0 with multiple tumors can substantially benefit from surgical treatment; thus, increasing the proportion of surgical treatment in this population can improve the overall prognosis.

## Figures and Tables

**Table 1:** Clinical characteristics of ICCAs with stage II before and after propensity score matching (PSM).

	Estimate	Before PSM			After PSM1			After PSM2		
		Single (n = 233)	Multiple (n = 434)	*p*	Single (n = 202)	Multiple (n = 202)	*p*	Single (n = 198)	Multiple (n = 198)	*p*
Age (Median)	65	65	65		65	65		65	63	
<70 y	436 (65.4)	153 (65.7)	283 (65.2)	0.906	134 (66.3)	133 (65.8)	0.916	130 (65.7)	143 (72.2)	0.158
≥70 y	231 (34.6)	80 (34.3)	151 (34.8)		68 (33.6)	69 (34.2)		68 (34.3)	55 (27.8)	
Gender				0.671			0.232			0.314
Female	351 (52.6)	120 (51.5)	231 (53.2)		109 (54.0)	97 (48.0)		109 (55.1)	99 (50.0)	
Male	316 (47.4)	113 (48.5)	203 (46.7)		93 (46.0)	105 (52.0)		89 (44.9)	99 (50.0)	
Race				0.192			0.796			0.495
White	502 (75.3)	169 (72.5)	333 (76.7)		148 (73.3)	153 (75.7)		148 (74.7)	154 (77.8)	
Black	62 (9.3)	20 (8.6)	42 (9.7)		18 (8.9)	18 (8.9)		17 (8.6)	11 (5.6)	
Other *	103 (15.4)	44 (18.9)	59 (13.6)		36 (17.8)	31 (15.3)		33 (16.7)	33 (16.7)	
Size				0.026			0.837			0.534
≤5 cm	189 (28.3)	80 (34.3)	109 (25.1)		75 (37.1)	77 (38.1)		72 (36.4)	78 (39.4)	
>5 cm	384 (57.6)	127 (54.5)	257 (59.2)		127 (62.9)	125 (61.9)		126 (63.6)	120 (60.6)	
unknown	94 (14.1)	26 (11.2)	68 (15.7)		0	0		0	0	
Treatment				<0.001			<0.001			1.000
No surgical procedure	424 (63.6)	114 (48.9)	310 (71.4)		89 (44.1)	163 (80.7)		90 (45.5)	90 (45.5)	
resection	243 (36.4)	119 (51.1)	124 (28.6)		113 (55.9)	39 (19.3)		108 (54.5)	108 (54.5)	
Survival Rate				<0.001			<0.001			0.079
1 year (%)		60.8	41.4		61.7	33.7		60.9	53.0	
3 years (%)		32.1	13.4		32.6	12.9		31.8	22.6	
5 years (%)		22.0	10.0		21.6	9.5		20.6	16.6	

*: American Indian/AK Native, Asian/Pacific Islander.

**Table 2:** Clinical characteristics of ICCAs with T2mN0M0 and stage IIIB before and after PSM.

	Before PSM			After PSM1			After PSM2		
	Stage ⅢB (n = 788)	Multiple (n = 434)	*p*	Stage ⅢB (n = 366)	Multiple (n = 366)	*p*	Stage ⅢB (n = 361)	Multiple (n = 361)	*p*
Age (Median)	63	65		64	65		64	65	
<70 y	562 (71.3)	283 (65.2)	0.027	236 (64.5)	236 (64.5)	1.000	233 (64.5)	236 (65.4)	0.815
≥70 y	226 (28.7)	151 (34.8)		130 (35.5)	130 (35.5)		128 (35.5)	125 (34.6)	
Gender			0.409			0.375			0.551
Female	400 (50.8)	231 (53.2)		180 (49.2)	192 (52.5)		180 (49.9)	188 (52.1)	
Male	388 (49.2)	203 (46.8)		186 (50.8)	174 (47.5)		181 (50.1)	173 (47.9)	
Race			0.368			1.000			0.926
White	631 (80.1)	333 (76.7)		285 (77.9)	285 (77.9)		282 (78.1)	285 (78.9)	
Black	62 (7.9)	42 (9.7)		33 (9.0)	33 (9.0)		34 (9.4)	31 (8.6)	
Other *	95 (12.1)	59 (13.6)		48 (13.1)	48 (13.1)		45 (12.5)	45 (12.5)	
Size			0.034			1.000			0.470
≤5 cm	231 (29.3)	109 (25.1)		109 (29.8)	109 (29.8)		117 (32.4)	108 (29.9)	
>5 cm	406 (51.5)	257 (59.2)		257 (70.2)	257 (70.2)		244 (67.6)	253 (70.1)	
unknown	151 (19.2)	68 (15.7)		0	0		0	0	
Treatment			0.114			0.473			0.460
No surgical procedure	551 (69.9)	322 (74.2)		247 (67.5)	256 (69.9)		261 (72.3)	252 (69.8)	
Surgery	237 (30.1)	112 (25.8)		119 (32.5)	110 (30.1)		100 (27.7)	109 (30.2)	
Survival Rate			0.857			0.907			0.699
1 year (%)	48.9	41.4		54.4	43.9		53.5	44.5	
3 years (%)	12.8	13.4		13.8	15.3		13.1	15.5	
5 years (%)	6.7	8.6		5.0	9.6		5.3	9.7	

*: American Indian/AK Native, Asian/Pacific Islander.

**Table 3:** Clinical characteristics of ICCAs with stage II stratified by gender before and after PSM.

	Female iCCA before PSM			Male iCCA before PSM			Female iCCA after PSM1			Male iCCA after PSM1			Female iCCA after PSM2			Male iCCA after PSM2		
	Single (n = 120)	Multiple (n = 231)	*p*	Single (n = 113)	Multiple (n = 203)	*p*	Single (n = 109)	Multiple (n = 97)	*p*	Single (n = 93)	Multiple (n = 105)	*p*	Single (n = 109)	Multiple (n = 99)	*p*	Single (n = 89)	Multiple (n = 99)	*p*
Age (Median)	65	64		65	65		65	65		66	65		61	60		75	75	
			0.693			0.555			0.868			1.000			0.125			0.608
<70 y	78 (65.0)	155 (67.1)		75 (66.4)	128 (63.1)		72 (66.1)	63 (64.9)		62 (66.7)	70 (66.7)		72 (66.1)	75 (75.8)		58 (65.2)	68 (68.7)	
≥70 y	42 (35.0)	76 (32.9)		38 (33.6)	75 (36.9)		37 (33.9)	34 (35.1)		31 (33.3)	35 (33.3)		37 (33.9)	24 (24.2)		31 (34.8)	31 (31.3)	
Race			0.562			0.094			0.602			0.930			0.483			1.000
White	84 (70.0)	174 (75.3)		85 (75.2)	159 (78.3)		76 (69.7)	73 (75.3)		72 (77.4)	80 (76.2)		76 (69.7)	74 (74.7)		72 (80.9)	80 (80.8)	
Black	15 (12.5)	24 (10.4)		5 (4.4)	18 (8.9)		13 (11.9)	11 (11.3)		5 (5.4)	7 (6.7)		13 (11.9)	7 (7.1)		4 (4.5)	4 (4.0)	
Other *	21 (17.5)	33 (14.3)		23 (20.4)	26 (12.8)		20 (18.3)	13 (13.4)		16 (17.2)	18 (17.1)		20 (18.3)	18 (18.2)		13 (14.6)	15 (15.2)	
Size			0.007			0.781			0.688			0.540			0.413			0.110
≤5 cm	40 (33.3)	45 (19.5)		40 (35.4)	64 (31.5)		40 (36.7)	33 (34.0)		35 (37.6)	44 (41.9)		40 (36.7)	31 (31.3)		32 (36.0)	47 (47.5)	
>5 cm	69 (57.5)	147 (63.6)		58 (51.3)	110 (54.2)		69 (63.3)	64 (66.0)		58 (62.4)	61 (58.1)		69 (63.3)	68 (68.7)		57 (64.0)	52 (52.5)	
unknown	11 (9.2)	39 (16.9)		15 (13.3)	29 (14.3)		0	0		0	0		0	0		0	0	
Treatment			<0.001			<0.001			<0.001			<0.001			0.724			0.728
No surgery procedure	61 (50.8)	162 (70.1)		53 (46.9)	148 (72.9)		50 (45.9)	79 (81.4)		39 (41.9)	84 (80.0)		50 (45.9)	43 (43.4)		40 (44.9)	47 (47.5)	
surgery	59 (49.2)	69 (29.9)		60 (53.1)	55 (27.1)		59 (54.1)	18 (18.6)		54 (58.1)	21 (20.0)		59 (54.1)	56 (56.6)		49 (55.1)	52 (52.5)	
Survival Rate			0.007			<0.001			0.010			<0.001			0.943			0.015
1 years (%)	60.7	46.8		60.9	35.3		59.4	40.2		64.4	27.6		59.4	60.6		62.8	45.5	
3 years (%)	28.7	15.9		35.8	10.5		28.8	15.5		37.1	10.5		28.8	28.0		35.4	17.2	
5 years (%)	16.5	10.7		23.0	6.2		15.4	10.4		23.3	4.1		15.4	18.4		21.2	9.4	

*: American Indian/AK Native, Asian/Pacific Islander.

**Table 4:** Variables Associated with Overall Survival Using Cox Proportional Hazards Model in iCCAs with stage II.

Variable	Cox Proportional Hazards Analysis			
	Univariate		Multivariate	
	HR (95% CI)	*p*-Value	HR (95% CI)	*p*-Value
Age				
<70 y	Referent	NA	NA	NA
≥70 y	1.374 (1.144–1.650)	0.001	1.208 (1.005–1.453)	0.044
Sex				
Female	Referent	NA	NA	NA
male	1.124 (0.943–1.340)	0.193	NA	NA
Race				
White	Referent	NA	NA	NA
Black	0.991 (0.725–1.355)	0.955	NA	NA
Other *	0.918 (0.716–1.177)	0.500	NA	NA
Size				
≤5 cm	Referent	NA	NA	NA
>5 cm	1.216 (1.006–1.470)	0.044	NA	NA
Treatment				
No surgery procedure	Referent	NA	NA	NA
Surgery treatment	0.284 (0.233–0.346)	<0.001	0.304 (0.248–0.372)	<0.001
Number of lesions				
Solitary	Referent	NA	NA	NA
Multiple	1.539 (1.276–1.855)	<0.001	1.236 (1.021–1.496)	0.030

Abbreviations: HR, hazard ratio; NA, not applicable. *: American Indian/AK Native, Asian/Pacific Islander.

## Data Availability

The datasets generated during and/or analyzed during the current study are available in the [SEER] repository, [https://seer.cancer.gov/seerstat] accessed on 20 April 2022.

## Supplementary Materials

The following supporting information can be downloaded at: https://www.mdpi.com/article/10.3390/jcm12020675/s1, Figure S1: Kaplan-Meier survival curve of T2sN0M0 vs. T2mN0M0 in iCCAs stratified by types of surgery; Table S1: Specific meaning of CS extension; Table S2: Clinical characteristics of ICCAs with stage Ⅱ stratified by age before and after PSM; Table S3: Clinical characteristics and OS of ICCAs with stage Ⅱ stratified by types of surgery.
