# Supplementary material for: Validation of the Prognostic Role for Surgical Treatment in Stage II Intrahepatic Cholangiocarcinoma: A SEER Population-Based Study
Source: J Clin Med. 2023 Jan 14;12(2):675. doi: 10.3390/jcm12020675 (PMC9863371; doi:10.3390/jcm12020675)
Supplement: Supplementary file 1 [file jcm-12-00675-s001.zip › Supplementary Table S1.pdf]

Supplementary Table S1: Specific meaning of CS extension

Code	Description
200	Single lesion in one lobe of liver<br>WITH intrahepatic vascular invasion
220	Single lesion in more than one lobe of liver (contiguous growth)<br>WITH vascular invasion
240	(200 or 220) + 140<br><br>Single lesion in one or more lobes of liver (contiguous growth)<br>WITH vascular invasion plus extension to gallbladder
250	Single tumor<br>WITH major vascular invasion: major branch(es) of portal or hepatic vein(s)<br>(See Note 2)
260	250 + 140<br>Single tumor with major vascular invasion plus extension to gallbladder
270	Stated as T2a with no other information on extension
300	Multiple (satellite) nodules/tumors in one lobe of liver<br>WITHOUT intrahepatic vascular invasion, including vascular invasion not stated
400	Multiple (satellite) nodules/tumors in one lobe of liver(<br>WITH intrahepatic vascular invasion
420	(300 or 400) + 140<br>Multiple (satellite) nodules/tumors in one lobe of liver<br>WITH or WITHOUT intrahepatic vascular invasion plus extension to gallbladder
450	Multiple (satellite) nodules/tumor(s)<br>WITH major vascular invasion: major branch(es) of portal or hepatic vein(s)<br>(See Note 2)
455	450 + 140<br>Multiple (satellite) nodules/tumor(s) with major vascular invasion plus extension to gallbladder
460	Multiple (satellite) nodules/tumors in more than one lobe of liver<br>Satellite nodules
465	460 + 140<br>Multiple (satellite) nodules/tumors in more than one lobe of liver plus extension to gallbladder
470	(460 or 465) + any of (250, 260, 450, 455)
475	Stated as T2b* with no other information on extension
520	More than one lobe involved by contiguous growth (single lesion)<br>WITH vascular invasion
580	Extrahepatic bile ducts
620	580 + (460 or 465)<br>Extrahepatic bile ducts plus satellite nodules in more than one lobe or multiple tumors with major vascular invasion
630	Major vascular invasion: major branch(es) of portal or hepatic vein(s)
631	Single tumor with major vascular invasion: major branch(es) of portal or hepatic vein(s)
632	Multiple tumor(s) with major vascular invasion: major branch(es) of portal or hepatic vein(s)

Code	Description
640	Direct extension/perforation of visceral peritoneum
650	Multiple (satellite) nodules/tumors in more than one lobe of liver or on surface of parenchyma Satellite nodules
655	Stated as T2b* with no other information on extension
660	Extension to hepatic artery or vena cava
665	660 + (460, 465, 470)
670	650 + 630
675	650 + (631 or 632)
700	Diaphragm
750	Lesser omentum
	Ligament(s):
	Coronary
	Falciform
	Hepatoduodenal
	Hepatogastric
	Round (of liver)
	Triangular
	Perforation of visceral peritoneum
	Parietal peritoneum
755	Lesser omentum Ligament(s):
	Coronary
	Falciform
	Hepatoduodenal
	Hepatogastric
	Round (of liver)
	Triangular
	Parietal peritoneum
760	(650 or 670) + any of [(640) or (660) or (700) or (750)]
800	Further contiguous extension:
	Pancreas
	Pleura
	Stomach
	Other contiguous extension

*the T category is based on AJCC 7 staging schema.
