# Supplementary material for: Validation of the Prognostic Role for Surgical Treatment in Stage II Intrahepatic Cholangiocarcinoma: A SEER Population-Based Study
Source: J Clin Med. 2023 Jan 14;12(2):675. doi: 10.3390/jcm12020675 (PMC9863371; doi:10.3390/jcm12020675)
Supplement: Supplementary file 1 [file jcm-12-00675-s001.zip › Supplementary Table S2.pdf]

Supplementary Table S2: Clinical characteristics of ICCAs with stage II stratified by age before and after PSM.

	**Age<70 iCCA before PSM**			**Age≥70 iCCA before PSM**			**Age<70 iCCA after PSM1**			**Age≥70 iCCA after PSM1**			**Age<70 iCCA after PSM2**			**Age≥70 iCCA after PSM2**		
	**Single (n=153)**	**Multiple (n=283)**	***p***	**Single (n=80)**	**Multiple (n=151)**	***p***	**Single (n=134)**	**Multiple (n=133)**	***p***	**Single (n=68)**	**Multiple (n=69)**	***p***	**Single (n=130)**	**Multiple (n=143)**	***p***	**Single (n=68)**	**Multiple (n=55)**	***p***
Age (Median)	61	59		76	75		61	60		75	75		61	60		75	75	
Gender			0.449			0.754			0.298			0.547			0.627			0.235
Female	78(51.0)	155(54.8)		42(52.5)	76(50.3)		72(53.7)	63(47.4)		37(54.4)	34(49.3)		72(55.4)	75(52.4)		37(54.4)	24(43.6)	
Male	75(49.0)	128(45.2)		38(47.5)	75(49.7)		62(46.3)	70(52.6)		31(45.6)	35(50.7)		58(44.6)	68(47.6)		31(45.6)	31(56.4)	
Race			0.142			0.866			0.861			0.685			0.770			0.419
White	108(70.6)	218(77.0)		61(76.3)	115(76.2)		98(73.1)	101(75.9)		50(73.5)	52(75.4)		98(75.4)	112(78.3)		50(73.5)	42(76.4)	
Black	14(9.2)	28(9.9)		6(7.5)	14(9.3)		13(9.7)	11(8.3)		5(7.4)	7(10.1)		12(9.2)	10(7.0)		5(7.4)	1(1.8)	
Other*	31(20.3)	37(13.1)		13(16.3)	22(14.6)		23(17.2)	21(15.8)		13(19.1)	10(14.5)		20(15.4)	21(14.7)		13(19.1)	23(21.8)	
**Size**			0.014			0.495			0.762			0.936			0.900			0.066
≤5cm	48(31.4)	59(20.8)		32(40.0)	50(33.1)		43(32.1)	45(33.8)		32(47.1)	32(46.4)		40(30.8)	43(30.1)		36(52.9)	35(63.6)	
>5cm	91(59.5)	177(62.5)		36(45.0)	80(53.0)		91(67.9)	88(66.2)		36(52.9)	37(53.6)		90(69.2)	100(69.9)		32(47.1)	20(36.4)	
unknow	14(9.2)	47(16.6)		12(15.0)	21(13.9)		0	0		0	0		0	0		0	0	
Treatment			<0.001			0.001			<0.001			<0.001			0.515			0.409
No surgery procedure	69(45.1)	193(68.2)		45(56.3)	117(77.5)		53(39.6)	102(76.7)		36(52.9)	61(88.4)		54(41.5)	65(45.5)		36(52.9)	25(45.5)	
surgery	84(54.9)	90(31.8)		35(43.8)	34(22.5)		81(60.4)	31(23.3)		32(47.1)	8(11.6)		76(58.5)	78(54.5)		32(47.1)	30(54.5)	
Survival Rate			<0.001			<0.001			<0.001			<0.001			0.176			0.224
1 years (%)	63.2	45.9		56.2	33.1		64.7	39.1		55.9	23.2		63.6	55.9		55.9	45.5	
3 years (%)	37.3	15.9		22.5	8.6		37.3	15.8		23.5	7.2		36.1	25.0		23.5	16.4	
5 years	20.8	9.1		18.0	7.7		19.5	8.3		18.5	4.8		18.0	13.3		18.5	14.3	

(%)

*: American Indian/AK Native, Asian/Pacific Islander
