# Supplementary material for: Validation of the Prognostic Role for Surgical Treatment in Stage II Intrahepatic Cholangiocarcinoma: A SEER Population-Based Study
Source: J Clin Med. 2023 Jan 14;12(2):675. doi: 10.3390/jcm12020675 (PMC9863371; doi:10.3390/jcm12020675)
Supplement: Supplementary file 1 [file jcm-12-00675-s001.zip › Supplementary Table S3.pdf]

Supplementary Table S3: Clinical characteristics and OS of ICCAs with stage II stratified by types of surgery.

	Wedge or segmental resection			Lobectomy			Extended lobectomy		
	Single (n=43)	Multiple (n=26)	*p*	Single (n=41)	Multiple (n=45)	*p*	Single (n=14)	Multiple (n=22)	*p*
Age (Median)	64	60		62	61		62	63	
<70y	31(72.1)	21(80.8)	0.418	29(70.7)	34(75.6)	0.614	8(59.1)	13(57.1)	1.000
≥70y	12(27.9)	5(19.2)		12(29.3)	11(24.4)		6(40.9)	9(42.9)	
Gender			0.122			0.289			0.644
Female	28(65.1)	12(46.2)		19(46.3)	26(57.8)		7(50.0)	14(63.6)	
Male	15(34.9)	14(53.8)		22(53.7)	19(42.2)		7(50.0)	8(36.4)	
Race			0.187			0.090			0.912
White	28(65.1)	22(84.6)		26(63.4)	37(82.2)		11(78.6)	17(77.3)	
Black	5(11.6)	2(7.7)		7(17.1)	2(4.4)		1 (7.1)	1 (4.5)	
Other*	10(23.3)	2(7.7)		8(19.5)	6(13.3)		2(14.3)	4(18.2)	
Size			0.879			0.951			0.446
≤5cm	19(44.2)	11(42.3)		14(34.1)	14(31.1)		3(21.4)	5(22.7)	
>5cm	24(55.8)	15(57.7)		26(63.4)	30(66.7)		10(71.4)	17(77.3)	
unknow	0	0		1(2.4)	1(2.2)		1(7.1)	0	
Survival Rate			0.110			0.603			0.382
1 years (%)	79.1	80.8		82.7	80.0		71.4	63.6	
3 years (%)	48.8	34.6		62.7	43.9		42.9	27.3	
5 years (%)	30.5	11.5		32.6	35.6		28.6	21.8	

*: American Indian/AK Native, Asian/Pacific Islander
