# Supplementary figures and images for: Validation of the Prognostic Role for Surgical Treatment in Stage II Intrahepatic Cholangiocarcinoma: A SEER Population-Based Study
Source: J Clin Med. 2023 Jan 14;12(2):675. doi: 10.3390/jcm12020675 (PMC9863371; doi:10.3390/jcm12020675)

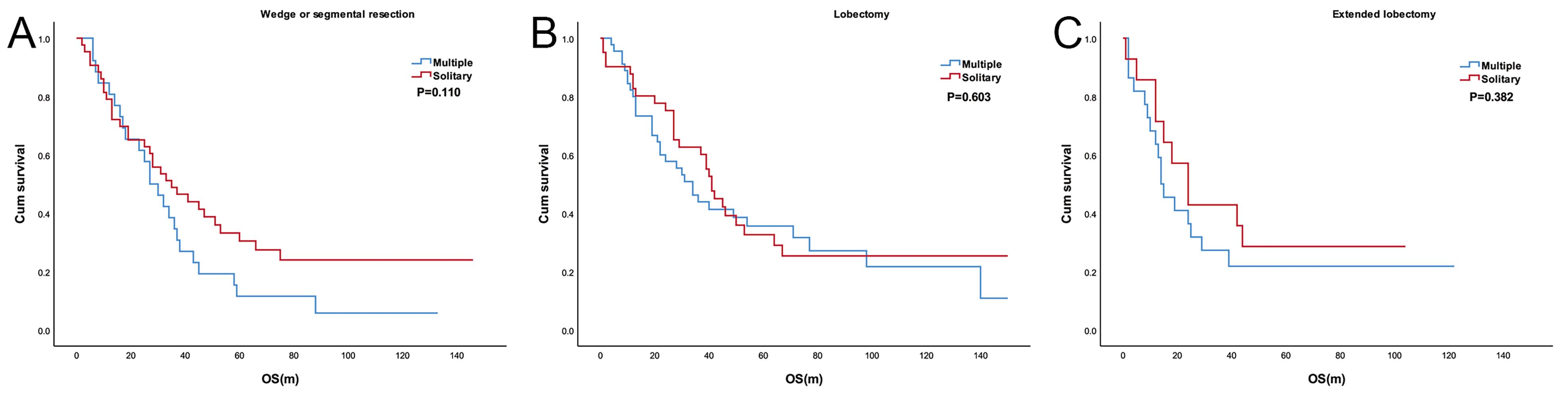

Supplement: Supplementary file 1 [file jcm-12-00675-s001.zip › Supplementary Fig S1.tif]
